# Enhancing quality of life in people with disordered eating using an online self-help programme

**DOI:** 10.1186/2050-2974-1-9

**Published:** 2013-03-11

**Authors:** Sau F Leung, Joyce LC Ma, Janice Russell

**Affiliations:** 1 School of Nursing, The Hong Kong Polytechnic University, Hung Hom, Kowloon, Hong Kong, China; 2 Department of Social Work, The Chinese University of Hong Kong, Shatin, New Territories, Hong Kong, China; 3 Discipline of Psychiatry, Faculty of Medicine, Sydney Medical School, University of Sydney, Sydney, NSW, 2006, Australia

**Keywords:** Quality of life, Eating disorders, Internet-based self-help programme

## Abstract

**Background:**

Eating disorders are serious mental illnesses that have a significant effect on afflicted individuals’ quality of life. Evidence has shown that they can be improved with treatment. Internet-based interventions are useful in engaging individuals with eating disorders in self-management and treatment. This study aimed primarily to identify the change in quality of life of individuals with disordered eating after participating in an open trial of an Internet-based self-help programme, and compared their quality of life at assessment with that of healthy controls. Factors affecting their quality of life were examined. Secondary outcomes related to symptom improvement were also reported.

**Methods:**

This study included 194 individuals with disordered eating and 50 healthy controls. The former group was recruited from eating disorder outpatient clinics and treatment units, as well as via information disseminated through various Internet websites, while the healthy controls were recruited from university student newspapers and university campuses. The Medical Outcomes Study Short Form Survey (SF-36v2) was used to assess participants’ quality of life. Other measures were used to assess their symptoms and motivational stages of change to recover from an eating disorder. The Wilcoxon signed ranks test and one-way repeated measures ANOVA were used to identify the change in quality of life of individuals with disordered eating from baseline to 1-, 3- and 6-month follow-ups. The Mann–Whitney *U* test was employed to compare the difference in quality of life between participants with disordered eating and the healthy controls. Spearman rank order correlations were performed to examine the factors associated with quality of life.

**Results:**

The participants with disordered eating had significantly poorer quality of life than the healthy controls in both physical and psychological domains. The factors associated with their poor quality of life included dieting behaviour, use of laxatives, severe eating disorder psychopathology, depression and anxiety. Over a six-month follow-up period, a significant number of participants engaged in self-help behaviours using the Internet-based programme. They experienced improvements in their quality of life, eating disorder psychopathology, depression severity, anxiety level and motivational stages of change.

**Conclusions:**

Internet-based self-help programmes have the potential to enhance quality of life in individuals with disordered eating and could be useful adjuncts to professional treatment.

## Background

Eating disorders are serious mental illnesses that impair cognitive function, judgment and emotional stability, and limit the life activities of the sufferers [1]. Such disorders have a significant impact on individuals’ quality of life, and can seriously affect their physical, social and psychological well-being [2,3]. The severity and chronicity of the illnesses are likely to exert negative effects on various life domains, such as physical functioning, performance at work or school, finances, interpersonal relationships, and social and role functioning [4-6]. Some factors in eating disorders have been reported to be associated with poorer quality of life, such as binge eating, vomiting, laxative use, use of diet pills, excessive exercise, severe psychopathology, extremes of BMI, and presence of psychiatric comorbidity [7-10].

Individuals with eating disorders have substantial impairment in quality of life, worse than those with other psychiatric disorders (e.g., alcohol abuse and somatoform disorder) [11], physical illnesses (e.g., angina and cystic fibrosis) [12], and healthy controls [2,13]. In a study comparing current and former eating disorder patients, current patients were shown to have poor quality of life in most domains, including sense of belonging, work/education, coping with disease-specific psychopathology, leisure activities, life skills, sense of purpose or meaning, financial situation, living condition, and particularly self-image and well-being [14]. Former eating disorder patients had better quality of life than current patients, but their ratings in different domains were only slightly above average. Another study found that former eating disorder patients had poorer quality of life than a normal reference group, even though they had recovered from their illnesses [2]. The findings of these studies underscore the impact of eating disorders on individuals’ quality of life, even following recovery.

There is some evidence showing that quality of life can be improved with face-to-face treatment [7,15], but this is limited to individuals who have access to professional services or who are willing to receive treatment. Internet-based interventions could be a viable alternative to reach out to individuals with eating disorders and engage them in self-management and treatment. Numerous European studies using a self-help approach have shown that Internet-based interventions can improve eating disorder symptoms, general psychopathology, self-esteem, depression and general life satisfaction [16-20]. However, little is known about their use in the Asia-Pacific region. It is important to replicate similar studies from the West and expand this innovative approach to therapy in the Asia-Pacific region, as it may have the potential to enhance the quality of life of individuals with eating disorders.

This study aimed primarily to i) identify the change in quality of life of individuals with disordered eating after using an Internet-based self-help programme developed in the Asia-Pacific region, ii) compare their quality of life with healthy controls at baseline assessment, and iii) identify the factors affecting their quality of life. Other secondary outcomes related to the improvement in eating disorder psychopathology, depression severity, anxiety level and motivational stages of change were also examined.

## Methods

### Design

This study was an open trial of an Internet-based self-help programme, the “Smart Eating” programme, developed in the Asia-Pacific region by the first author. A randomised controlled design was not used due to the difficulty of recruiting a large number of participants with eating disorders. This difficulty can be related to the ego-syntonic nature of the disorders, ambivalence toward treatment and poor acceptability of treatments [21,22]. The study was conducted in Hong Kong and Sydney, and was approved by the University of Sydney Human Research Ethics Committee and the relevant health service ethics committees. Participation in the study was voluntary. All participants were provided with a detailed information sheet when they logged into the self-help programme on the Internet. They were informed that further explanation of the study could be obtained from the investigators, either through direct personal contact or by e-mail. Written consent was obtained from each participant online through the self-help programme, and they were informed that they could withdraw from the study at any time.

Participants were required to complete an online registration in the self-help programme, and to choose their own usernames and passwords to log in to the programme. The online registration form elicited demographic information including gender, date of birth, body weight, body height, nationality, country of current residence, e-mail address, marital status, educational attainment, occupation, health history and treatments related to eating disorders, and assessment scores of the SCOFF questionnaire and EDE-Q, 5th edition (EDE-Q5). For security purposes, the demographic information and data provided by the users were protected by password and a special Web maintenance service.

The SCOFF questionnaire is a 5-item quick screening tool that addresses the core features of anorexia nervosa and bulimia nervosa [23]. A score of 2 or above in the SCOFF questionnaire indicates a high risk of having an eating disorder. The EDE-Q is a 28-item self-report questionnaire that focuses on symptoms that occurred within the previous 28 days [24]. Some items in the EDE-Q identify the behavioural features of eating disorders in terms of frequency or days the behaviour has occurred, and others assess the severity of eating disorder symptoms on four subscales: dietary restraint, eating concern, weight concern, and shape concern. The items on each subscale are rated on a 7-point rating scale (0–6). A mean score of 4–6 on any subscale is interpreted as meaning a greater likelihood of clinical severity.

#### Assessment questionnaires

The Medical Outcomes Study Short Form Survey (SF-36 version 2) was administered through the Smart Eating programme to assess the participants’ quality of life. It is the most widely used generic quality of life measure for individuals with eating disorders [12]. It contains 36 questions on eight scales to measure physical functioning, physical role functioning, bodily pain, general health, vitality, social functioning, emotional role functioning, and mental health [25]. The score of each scale ranges from 0–100, with higher scores indicating a better state of health. The eight scales can be summarised into the Physical Component Summary scale (PCS) and the Mental Component Summary scale (MCS).

In a large U.S. population study, SF-36 version 2 was identified to have high internal consistency reliability coefficients for all scales (well above 0.8) [25]. The SF-36 can be used to assess functional capacity and well-being across a wide variety of medical and psychiatric conditions, to monitor changes in functioning over time, and to evaluate treatment options [26]. The measure has been adapted for use in numerous large-scale studies and across different healthcare settings in Australia since 1992. In a random sample of 855 subjects from the general population, the SF-36 was reported to have good internal consistency reliability for all scales (Cronbach’s alpha = 0.81-0.92) and good construct validity in factor analysis, supporting the accuracy of both the physical and mental health dimensions [27]. In the first Australian national household panel survey involving 13,055 respondents, SF-36 was shown to be psychometrically sound, with good internal consistency, discriminant validity and high reliability across its eight scales [28]. The psychometric properties of SF-36 were further established in Australia from the data drawn from five studies with 41,338 participants aged 45 to 97 years in the Dynamic Analyses to Optimise Ageing (DYNOPTA) project [29]. These results confirmed the structural validity and good internal consistency reliability of SF-36, and were comparable with those found in younger Australian and international samples.

Other health assessment questionnaires offered by the Smart Eating programme include the Eating Disorder Examination Questionnaire (EDE-Q5) [24], the Eating Disorder Inventory-3 (EDI-3) [30], the Beck Depression Inventory (BDI-II) [31], the Beck Anxiety Inventory (BAI) [32], and the Motivational Stages of Change for Adolescents Recovering from an Eating Disorder (MSCARED) [33], which could be administered to both adolescents and adults in this study. EDE-Q5 is different from EDI-3, since EDE-Q5 measures the behavioural features of eating disorders, dietary restraint and concerns over eating, weight and shape [24], whereas the EDI-3 assesses the attitudes, behaviours and psychological traits of individuals with eating disorders [30]. Permission was obtained from the developers or authorities for online use of the questionnaires. All questionnaires demonstrated good psychometric properties in terms of internal consistency reliability [34-37], test-retest reliability [30,34-36,38], convergent validity [37], and concurrent validity [38].

Higher scores on the EDE-Q5, EDI-3, BDI-II, and BAI indicate more severe eating disorder psychopathology and associated psychological symptoms. Higher scores on the SF-36 and MSCARED indicate better perceived quality of life and higher motivation to change disordered eating behaviour.

### Participants

The participants were recruited by the first author. Those with disordered eating were recruited through personal approach or promotional leaflets distributed in two outpatient eating disorder clinics and two eating disorder treatment units, and information disseminated through various Internet websites. The healthy controls were recruited from two universities through university student newspapers and the on-campus distribution of promotional leaflets. Individuals were able to participate in the study if they were aged 16 to 50 years old (male or female) and identified themselves as healthy controls or suffering from an eating disorder as shown by the assessment scores of the SCOFF questionnaire or the EDE-Q5.

Participants who successfully registered in the programme were invited via e-mail to complete six self-assessment questionnaires available in the self-help programme. After completing this baseline assessment, they received an e-mail with instructions on using the programme. The participants were reminded via e-mail to complete the six self-assessment questionnaires at the 1- and 3-month follow-up periods, as well as at the 6-month follow-up if possible. Participants with disordered eating were also followed up monthly through e-mail correspondence. The e-mails were standardised, enquiring about individual progress in using the programme and encouraging them to continue.

### Materials

#### The smart eating self-help programme

The Smart Eating self-help programme comprises several components relating to healthy eating, family education, health assessment, motivation enhancement, self-help strategies, and psychological health promotion. The motivation enhancement component encourages individuals’ motivation to change and recover from an eating disorder. The self-help strategies introduced in the programme help individuals restore healthy weight; normalise eating patterns; manage dieting, bingeing, and purging; challenge negative automatic thoughts and thinking errors; develop problem-solving abilities; and prevent relapse. The psychological health promotion component covers strategies for coping with stress, body image alteration, low self-esteem, depression, and anxiety. The content of the programme incorporates expert input from eating disorder specialists and is consistent with treatment recommendations for eating disorders from different eating disorder practice guidelines, including those of the American Psychiatric Association (APA) [39], the National Institute for Clinical Excellence (NICE) [40] and the Royal Australian and New Zealand College of Psychiatrists [41,42]. Before its launch, the programme was examined in a usability test on 15 female inpatients to demonstrate its usefulness, satisfactory information quality and good interface quality.

To acquire the maximum benefit from the Smart Eating programme, participants with disordered eating were recommended to go through its individual components in the following pattern as prescribed in the programme:

1. Browse through the information on healthy eating

2. Solicit support from family members to overcome an eating disorder, and ask them to view the information about family education

3. Complete all health assessment questionnaires at baseline

4. Work through the worksheets on motivational enhancement

5. Follow the steps in the self-help strategies to overcome eating disorders

6. Use different strategies to promote psychological health

7. Complete all health assessment questionnaires again at 1-, 3- and 6-month follow-up intervals to monitor personal progress

The healthy controls in this study had access limited to the programme components of healthy eating, family education and health assessment. They could use the programme to find out whether they had an eating disorder or an associated psychological problem (e.g. anxiety or depression). Participants with disordered eating had access to all components of the programme, and used it to learn and apply various self-help strategies and perform online self-assessments to monitor their progress in eating disorder psychopathology, motivational stage of change, and psychological health. They also used the programme privately at a time and pace convenient to themselves. A tracking system was implemented to determine compliance with the programme regarding health assessment, motivation enhancement, self-help strategies and psychological health promotion.

### Data analysis

The data were analysed using the Predictive Analytics Software (PASW 17.0) statistical package. Descriptive analysis was performed to identify participants’ demographic characteristics. The Wilcoxon signed ranks test and one-way repeated measures ANOVA were used to identify changes in the quality of life of individuals with disordered eating from baseline to the 1-, 3- and 6-month follow-ups. The Mann–Whitney *U* test was employed to compare the differences in quality of life between participants with disordered eating and the healthy controls. Spearman rank order correlations were performed to examine the factors affecting individuals’ quality of life.

## Results

### Participant characteristics

**Figure 1:**
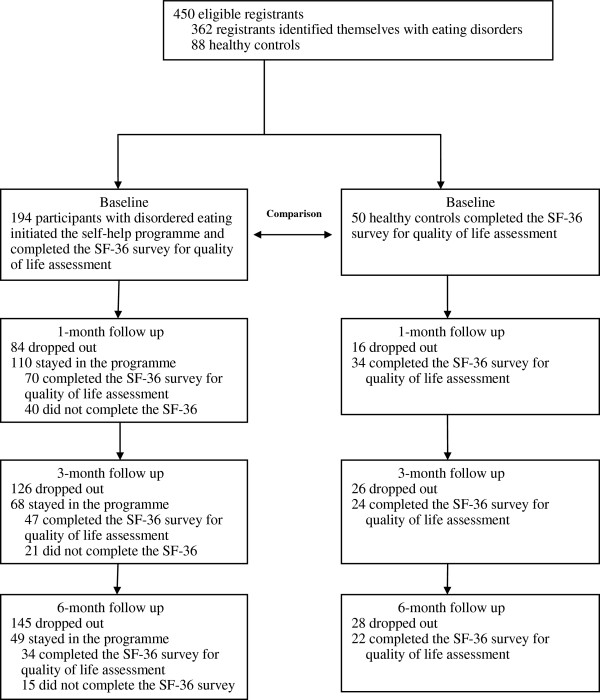
Flow of participants in the Smart Eating self-help programme.

A total of 362 individuals who identified themselves as having an eating disorder and 88 healthy controls registered in the programme from August 2006 to May 2012. The former group is referred to as the participants with disordered eating in this study. Among them, 194 (53.6%) participants with disordered eating and 50 (56.8%) healthy controls initiated their baseline health assessment with the Smart Eating programme, and their findings are reported in this paper (Figure 1). Reminders were sent to those individuals who did not initiate the programme, but they did not respond.

The 194 participants with disordered eating comprised 191 females and 3 males. Their ages ranged from 16 to 50 years (mean age 26.9). Their mean body mass index (BMI) was 21.25 (SD 5.99; range 13.05-57.81). Among them, 82 (42.3%) were not receiving any specialist treatment for eating disorders, and 112 (57.7%) were receiving treatment. The treatment was provided by psychiatrists/medical practitioners (86/194, 44.3%), psychologists/counsellors (44/194, 22.7%), dieticians (28/194, 14.4%) or a mental health nurse specialist (1/194, 0.5%). Some of the participants reported taking medication (50/194, 25.8%), and a few were using Chinese herbal medicine (1/194, 0.5%), acupuncture (1/194, 0.5%), naturopathy (1/194, 0.5%), or nutritional supplements (2/194, 1%) in their treatment. Among the healthy controls, there were 45 females and 5 males. Their ages ranged from 17 to 48 years (mean age 24.7). Their mean BMI was 20.7 (SD 3.14; range 16.2-31.3). Most of the participants were Australian or Hong Kong Chinese. A majority of them had a secondary education or higher. Table 1 shows detailed demographic information.

**Table 1:** Demographic characteristics of participants (n=244)

**Variables**	**Participants with disordered eating (n=194)**		**Healthy controls (n=50)**	
	**Number**	**Percentage**	**Number**	**Percentage**
Gender				
Female	191	98.5	45	90
Male	3	1.5	5	10
Educational level				
No schooling	3	1.5	0	0
Secondary	62	32	4	8
Tertiary	128	66	45	90
Unknown	1	0.5	1	2
Marital status				
Single	134	69.1	45	90
Married	32	16.5	5	10
Cohabiting	22	11.3	0	0
Divorced	5	2.6	0	0
Widowed	1	0.5	0	0
Employment status				
Full-time employment	92	47.4	11	22
Unemployed	13	6.7	0	0
Homemaker	8	4.1	0	0
Student	81	41.8	39	78
Nationality/National group				
Australian	142	73.2	3	6
Chinese	12	6.2	41	82
Other Asian	9	4.6	0	0
European	12	6.2	2	4
New Zealander	10	5.1	1	2
Canadian	4	2.1	0	0
American	4	2.1	2	4
Others	1	0.5	1	2

### Online health assessment scores of the participants

The eating disorder psychopathology of the participants was examined by the online self-administered SCOFF questionnaire and the Eating Disorder Examination Questionnaire (EDE-Q 5^th^ edition) when they registered in the Smart Eating programme. According to the assessment scores, 191 participants with disordered eating scored at least 2 on the SCOFF questionnaire or had at least one subscale score on the EDE-Q5 falling in the clinically significant range (≥ 4). The three remaining participants had non-significant assessment scores but reported participating in treatment for their eating disorders, and two had low BMI (15.59 and 16.53). Thus, all 194 participants with disordered eating had a clinically significant eating disorder based on their SCOFF questionnaire/EDE-Q5 scores or their treatment for an eating disorder. The participants with disordered eating had significantly higher mean scores in the SCOFF questionnaires (3.36) and the Eating Disorder Examination Questionnaire (≥4 for each subscale) than the healthy controls (Table 2). At baseline assessment, the former group also had significantly higher scores on EDI-3, BDI-II, and BAI, indicating more severe eating disorder psychopathology, depressive symptoms and anxiety level than the latter group.

**Table 2:** **Comparison of health assessment scores of participants with disordered eating and healthy controls at baseline using the Mann–Whitney *U *** **test**

**Variables**	**Participants with disordered eating (n=194)**		**Healthy controls (n=50)**		**Z**	**P value**
	**(M)**	**(SD)**	**(M)**	**(SD)**		
**SCOFF score**	3.36	0.95	0.63	0.83	−10.45	<0.001
**EDE-Q5 (global score)**	4.47	0.97	0.92	0.78	−10.58	<0.001
*Dietary restraint*	4.08	1.24	0.55	0.75	−10.44	<0.001
*Eating concern*	4.15	1.25	0.42	0.59	−10.61	<0.001
*Shape concern*	4.91	1.15	1.70	1.27	−9.89	<0.001
*Weight concern*	4.66	1.24	1.25	1.20	−9.94	<0.001
SF-36v2*	52.40	16.14	75.82	11.89	−8.14	<0.001
***PCS***	65.35	18.20	79.26	11.74	−4.97	<0.001
*Physical functioning*	84.66	18.10	89.80	16.57	−2.32	0.021
*Physical role functioning*	67.05	28.75	85.64	17.46	−4.22	<0.001
*Bodily pain*	64.71	23.81	82.12	20.95	−4.66	<0.001
*General health*	44.98	23.42	59.48	19.26	−4.02	<0.001
***MCS***	39.45	18.27	72.38	16.05	−8.74	<0.001
*Vitality*	31.18	19.25	57.72	18.04	−7.38	<0.001
*Social functioning*	40.74	26.17	83.58	19.32	−8.59	<0.001
*Emotional rolefunctioning*	45.45	26.43	80.52	19.69	−7.55	<0.001
*Mental health*	40.44	18.60	67.70	20.18	−7.39	<0.001
**EDI-3**	16.29	4.11	6.70	2.78	−9.99	<0.001
*Drive for thinness*	22.42	5.40	5.80	5.48	−9.89	<0.001
*Bulimia*	16.50	9.43	3.74	3.52	−7.86	<0.001
*Body dissatisfaction*	29.75	8.46	14.15	8.90	−8.15	<0.001
*Low self-esteem*	15.19	5.49	5.35	3.59	−8.99	<0.001
*Personal alienation*	15.06	5.88	6.00	4.95	−8.02	<0.001
*Interpersonal insecurity*	13.96	5.86	6.28	4.33	−7.32	<0.001
*Interpersonal alienation*	12.53	5.46	5.74	3.86	−7.18	<0.001
*Interoceptive deficits*	19.17	7.67	6.30	5.65	−8.56	<0.001
*Emotional dysregulation*	10.22	6.64	4.61	3.99	−5.51	<0.001
*Perfectionism*	14.51	6.20	7.09	4.58	−6.86	<0.001
*Asceticism*	13.78	5.74	4.17	2.74	−9.09	<0.001
*Maturity fears*	12.37	7.33	11.20	5.10	−0.71	0.478
**BDI-II**	32.51	13.28	7.19	7.94	−9.41	<0.001
**BAI**	19.66	12.10	4.71	4.05	−8.82	<0.001
**MSCARED #**	2.89	0.98	0	0	−11.00	<0.001

SCOFF, The SCOFF Questionnaire; EDE-Q5, Eating Disorder Examination Questionnaire version 5.

SF-36v2*, Mean score of physical and mental health domains of the 36-item Short Form Health Survey version 2.

PCS, SF-36 Physical Component Summary scale; MCS, SF-36 Mental Component Summary scale.

EDI-3, Eating Disorder Inventory version 3; BDI-II, Beck Depression Inventory version 2.

BAI, Beck Anxiety Inventory.

MSCARED, The Motivational Stages of Change for Adolescents Recovering from an Eating Disorder.

# Motivational stages of change.

0=Not applicable, 1=Pre-contemplation, 2=Contemplation, 3=Preparation, 4=Action, 5=Maintenance, 6=Recovery.

SF-36 measured the quality of life of the participants. Those with disordered eating were identified as having significantly poorer quality of life than the healthy controls in both physical (physical functioning, physical role functioning, bodily pain and general health) and mental health domains (vitality, social functioning, emotional role functioning and mental health). Their quality of life in the mental health domains was also worse than in the physical health domains. This was also shown from the lower score in the Mental Component Summary scale (MCS) than the Physical Component Summary scale (PCS) of SF-36. Table 2 compares the differences in all the health assessment scores at baseline between the participants with disordered eating and the healthy controls.

### Factors affecting the quality of life of participants with disordered eating

To identify the factors associated with poor quality of life in participants with disordered eating, Spearman rank order correlations were performed among various variables concerning eating disorder psychopathology, depression severity, anxiety level, motivational stages of change and quality of life. The findings revealed that quality of life (SF-36 score; mean scores of both physical and mental health domains) had a significant negative correlation with dieting behaviour (r_s=_-0.165, p<0.05) and use of laxatives (r_s=_ -0.167, p=<0.05) (Table 3). Also, quality of life was significantly affected by the increase in eating disorder psychopathology, depression severity and anxiety level, as shown from the negative correlation between SF-36 scores and those on the SCOFF questionnaire (r_s_= −0.204, p<0.01), Global EDE-Q5 (r_s_= −0.428, p<0.01), EDI-3 (r_s_= −0.573, p<0.01), BDI-II (r_s_= −0.715, p<0.01) and BAI (r_s_= −.592, p<0.01) (Table 3). However, there was a significant positive correlation between individuals’ motivational stages of change and the quality of life domains of physical function (r_s_= 0.168, p<0.05), general health (r_s_= 0.208, p<0.01), vitality (r_s_= 0.209, p<0.01) and mental health (r=0.146, p<0.05).

**Table 3:** Spearman’s rank-order correlations between quality of life and eating disorder psychopathology, eating disorder behaviours, depression severity, anxiety level and motivational stages of change

	**SF-36 v2**	***Physical functioning***	***Physical role functioning***	***Bodily pain***	***General health***	***Vitality***	***Social functioning***	***Emotional role functioning***	***Mental health***
**SCOFF**	−0.204**	**−0.002**	−0.141*	**−0.129**	−0.154*	**−0.120**	−0.204**	−0.189**	−0.204**
**Global EDE-Q5**	−0.428**	−0.254**	−0.276**	−0.239**	−0.327**	−0.341**	−0.350**	−0.323**	−0.418**
***Dieting***	−0.165*	**−0.069**	**−0.066**	**−0.118**	−0.149*	−0.170*	**−0.118**	**−0.104**	−0.208**
***Vomiting***	**−0.036**	**0.019**	**−0.002**	**−0.051**	**−0.025**	**−0.012**	**−0.032**	**−0.088**	**−0.040**
***Bingeing***	**0.084**	**0.120**	**0.109**	0.190*	0.185*	**0.107**	**0.051**	−0.153*	**−0.038**
***Laxative misuse***	−0.167*	−0.172*	**−0.109**	−0.249**	**−0.094**	**−0.060**	**−0.061**	**−0.122**	**−0.113**
***Compulsive exercise***	**−0.053**	0.256**	**0.017**	−0.163*	**0.036**	**0.048**	−0.262**	**−0.059**	**−0.098**
**EDI-3**	−0.573**	−0.266**	−0.354**	−0.295**	−0.421**	−0.476**	−0.424**	−0.472**	−0.602**
**BDI-II**	−0.715**	−0.375**	−0.437**	−0.314**	−0.537**	−0.611**	−0.553**	−0.561**	−0.742**
**BAI**	−0.592**	−0.292**	−0.393**	−0.409**	−0.372**	−0.379**	−0.482**	−0.493**	−0.567**
**MSCARED**	**0.113**	0.168*	**0.018**	**0.071**	0.208**	0.209**	**0.017**	**−0.037**	0.146*

*Significance P<0.05, ** Significance P<0.01.

SF-36v2, The 36-item Short Form Health Survey version 2; SCOFF, The SCOFF Questionnaire.

EDE-Q5, Eating Disorder Examination Questionnaire version 5; EDI-3, Eating Disorder Inventory version 3.

BDI-II, Beck Depression Inventory version 2; BAI, Beck Anxiety Inventory.

MSCARED, The Motivational Stages of Change for Adolescents Recovering from an Eating Disorder.

### Change in the quality of life of participants with disordered eating in this open trial study

The tracking system of the Smart Eating programme found that 194 participants with disordered eating engaged in various self-help behaviours, such as completing at least one baseline health assessment (177/194, 91.2%), working through motivation enhancement exercises (108/194, 55.7%), learning how to improve body image (91/194, 46.9%), and using self-help strategies to overcome eating disorders (63/194, 32.5%). Other self-help behaviours included identifying strategies for overcoming depression (55/194, 28.4%), managing anxiety (53/194, 27.3%), coping with stress (50/194, 25.8%), and boosting self-esteem (47/194, 24.2%).

Seventy participants (36.1%) used the programme for at least 1 month. The Wilcoxon signed ranks test identified significant improvement in their quality of life (Z= −2.581, p<0.05), particularly in the domains of bodily pain (Z= −2.167, p<0.05), general health (Z= −2.623, p<0.01), social functioning (Z= −2.475, p<0.05) and emotional role functioning (Z= −2.382, p<0.05) from baseline to 1-month follow up. The improvement in quality of life continued at the 3-month follow up for 47 participants (F=6.532, p<0.01), who also experienced significant improvements in their general health (F=5.655, p<0.01), social functioning (F=10.69, p<0.001), emotional role functioning (F=11.877, P<0.001) and mental health (F=7.746, p<0.01), as shown by the result of the one-way repeated measures ANOVA. At the 6-month follow up, 34 participants were still using the programme. Table 4 shows that these participants had significant improvement in their quality of life, eating disorder psychopathology, depression level and motivational stages of change from baseline assessment to the 1-month, 3-month and 6-month follow-ups. They had significant increases in their SF-36 and MSCARED scores, and significant decreases in their EDE-Q5, EDI-3 and BDI-II scores.

**Table 4:** Comparison of health assessment scores of participants with disordered eating from baseline to 1-, 3- and 6-month follow-ups using one-way repeated measures ANOVA

**N=34**	**Time 1 (baseline)**	**Time 2 (1-month FU)**	**Time 3 (3-month FU)**	**Time 4 (6-month FU)**	**F**	**Partial eta squared**	**P value**
	**Mean ± s.d.**	**Mean ± s.d.**	**Mean ± s.d.**	**Mean ± s.d.**			
SF-36v2*	45.60±15.84	50.23±19.58	55.72±22.41	59.23±24.27	4.93	0.323	**0.007**
***PCS***	59.67±21.05	61.34±24.38	64.15±24.17	68.95±25.34	2.30	0.182	0.097
*Physical functioning*	79.71±21.46	79.56±25.74	82.06±23.59	82.35±20.12	0.26	0.025	0.852
*Physical role functioning*	57.32±33.57	57.18±33.87	62.94±29.68	69.29±32.93	1.80	0.148	0.168
*Bodily pain*	59.71±25.72	63.35±25.93	61.38±29.90	70.38±29.31	2.01	0.162	0.134
*General health*	41.94±29.26	45.26±28.59	50.24±30.17	53.76±31.06	2.51	0.196	0.077
***MCS***	31.54±13.81	39.12±18.78	47.28±24.18	49.51±26.70	8.40	0.448	**<0.001**
*Vitality*	27.18±20.58	32.29±22.36	36.00±27.29	38.53±27.65	2.61	0.202	0.069
*Social functioning*	29.38±21.17	38.94±26.60	49.71±31.25	50.03±32.03	5.78	0.359	**0.003**
*Emotional role functioning*	34.29±22.51	46.56±23.79	56.65±24.95	59.76±32.34	9.40	0.476	**<0.001**
*Mental health*	35.29±14.92	38.68±17.42	46.76±23.25	49.71±23.90	6.40	0.382	**0.002**
**Global EDE-Q5**	4.63±0.83	3.99±1.25	3.34±1.53	3.26±1.72	12.52	0.540	**<0.001**
**EDI-3**	17.44±3.44	16.08±4.67	14.45±5.68	13.43±5.92	11.05	0.517	**<0.001**
**BDI-II**	37.29±12.48	31.71±15.33	26.26±16.60	23.82±17.43	8.53	0.452	**<0.001**
**BAI**	20.63±10.47	19.34±11.99	18.17±13.53	16.94±14.09	1.07	0.091	0.377
**MSCARED**	3.18±0.90	3.53±1.05	3.91±1.22	4.03±1.38	4.57	0.307	**0.009**

FU, follow up; s.d., standard deviation.

SF-36v2*, Mean score of physical and mental health domains of the 36-item Short Form Health Survey version 2.

PCS, SF-36 Physical Component Summary scale; MCS, SF-36 Mental Component Summary scale.

EDE-Q5, Eating Disorder Examination Questionnaire version 5; EDI-3, Eating Disorder Inventory version 3.

BDI-II, Beck Depression Inventory version 2; BAI, Beck Anxiety Inventory.

MSCARED, The Motivational Stages of Change for Adolescents Recovering from an Eating Disorder.

### Intention-to-treat analysis

In view of the number of participants with disordered eating who did not complete the follow-up health assessment and self-help programme, an intention-to-treat analysis was also performed. This type of analysis is used for clinical trial data in which the outcomes of all participants, including those who drop out prematurely, are included in the analysis. The last observation carried forward (LOCF) method was used, in which the missing values of the outcome variables are replaced by the most recent known values before the participants were lost to follow up [43]. This is based on the rationale that participants with eating disorders are unlikely to improve further in the absence of treatment.

The intention-to-treat analyses revealed significant increases in their SF-36 and MSCARED scores from baseline to 1-, 3- and 6-month follow-ups and significant decreases in their EDE-Q5, EDI-3 and BDI-II scores (Table 5). These findings indicate a significant improvement in their quality of life revealed by the SF-36, particularly in their bodily pain, general health, social functioning, emotional role functioning and mental health. Also there was a significant reduction in their eating disorder psychopathology as measured by the EDE-Q5 and the EDI-3. In addition, there were significant decreases in their depression levels, assessed by the BDI-II, along with an increase in their motivational stages of change, measured by the MSCARED. Although the BAI scores from baseline to 6-month follow-up showed a decrease in participants’ anxiety levels, the change was non-significant.

**Table 5:** Comparison of health assessment scores of participants with disordered eating from baseline assessment to 1-, 3- and 6-month follow-ups in the intention-to-treat analyses using one-way repeated measures ANOVA

**N=194**	**Time 1 (baseline)**	**Time 2 (1-month FU)**	**Time 3 (3-month FU)**	**Time 4 (6-month FU)**	**F**	**Partial eta squared**	**P value**
	**Mean ± s.d.**	**Mean ± s.d.**	**Mean ± s.d.**	**Mean ± s.d.**			
SF-36v2*	52.40±16.14	53.87±17.19	55.00±18.06	55.62±18.53	5.29	0.077	**0.002**
***PCS***	65.35±18.20	66.48±19.16	67.07±19.55	67.92±19.77	3.33	0.050	**0.021**
*Physical functioning*	84.66±18.10	84.38±20.17	84.87±19.60	84.92±18.93	0.31	0.005	0.816
*Physical role functioning*	67.05±28.75	68.18±29.08	69.48±28.07	70.60±28.53	2.57	0.039	0.056
*Bodily pain*	64.71±23.81	66.68±23.50	66.13±25.17	67.79±25.06	2.89	0.043	**0.037**
*General health*	44.98±23.42	46.68±23.45	47.80±23.92	48.37±24.15	4.04	0.060	**0.008**
***MCS***	39.45±18.27	41.30±19.63	42.98±20.77	43.35±21.37	5.97	0.086	**0.001**
*Vitality*	31.18±19.25	32.03±20.33	33.18±21.22	33.65±21.43	2.63	0.040	0.051
*Social functioning*	40.74±26.17	43.57±27.43	45.33±28.22	45.32±28.29	4.87	0.071	**0.003**
*Emotional role functioning*	45.45±26.43	48.03±26.38	50.18±26.67	50.76±28.21	5.30	0.077	**0.002**
*Mental health*	40.44±18.60	41.57±19.31	43.22±20.63	43.69±20.84	5.09	0.074	**0.002**
**Global EDE-Q5**	4.47±0.97	4.16±1.19	4.00±1.33	3.97±1.39	14.27	0.194	**<0.001**
**EDI-3**	16.29±4.10	15.81±4.39	15.48±4.68	15.29±4.80	9.40	0.131	**<0.001**
**BDI-II**	32.51±13.28	30.57±13.76	29.44±14.31	28.99±14.62	9.07	0.127	**<0.001**
**BAI**	19.66±12.10	19.10±11.94	18.87±12.36	18.64±12.49	1.25	0.020	0.292
**MSCARED**	3.22±0.89	3.36±0.92	3.43±0.98	3.44±1.05	6.41	0.093	**<0.001**

FU, follow up; s.d., standard deviation.

SF-36v2*, Mean score of physical and mental health domains of the 36-item Short Form Health Survey version 2.

PCS, SF-36 Physical Component Summary scale; MCS, SF-36 Mental Component Summary scale.

EDE-Q5, Eating Disorder Examination Questionnaire version 5; EDI-3, Eating Disorder Inventory version 3.

BDI-II, Beck Depression Inventory version 2; BAI, Beck Anxiety Inventory.

MSCARED, The Motivational Stages of Change for Adolescents Recovering from an Eating Disorder.

## Discussion

The Smart Eating programme engaged a significant number of individuals with disordered eating in self-help behaviour and also reached out to a number of those not receiving eating disorder treatment. Over a period of time using the programme, the participants saw an improvement in their quality of life, particularly in general health, social functioning, emotional role functioning and mental health as demonstrated by the increase in domain scores from baseline to the 1-, 3- and 6-month follow-ups. Improvement was also identified in participants’ eating disorder psychopathology, depression severity, anxiety level and motivational stages of change. There were significant decreases in the EDE-Q5, EDI-3 and BDI-II scores and increases in their MSCARED scores. The intention-to-treat analyses produced similar results to the completer analyses at the 3- and 6-month follow-ups. The self-help programme appears to have the potential to enhance quality of life in individuals with disordered eating by reducing their eating disorder symptoms, depression and anxiety, and improving their motivational stages of change. A significant number of participants in this study (112/194, 57.7%) were already in treatment, but they also used the programme. This implies that the programme could be a useful adjunct to their professional treatment.

As expected, there was a significant difference in the clinical features between participants with disordered eating and the healthy controls. The difference was recognised in their assessment scores on the SCOFF questionnaire, EDE-Q5, EDI-3, BDI-II, BAI and SF-36, which showed that participants with eating disorders had severe eating disorder symptoms, depression and anxiety, resulting in poorer quality of life. Their quality of life was much worse than that of healthy controls in all the domains studied in the SF-36. Consistent with earlier findings, this study confirmed several associated factors affecting individuals’ quality of life, including dieting, use of laxatives, severe eating disorder psychopathology and the existence of psychiatric comorbidity such as anxiety and depression [7-9]. A significant negative correlation was observed between quality of life and these factors in the study sample. Similar to the findings of other studies, eating disorders caused more impairment in the mental health domains of the participants’ quality of life than in the physical health domains, and the impairment was more noticeable in vitality, mental health and social functioning in the SF-36 [2,44-46].

This study had several limitations. One was the convenience (non-random pragmatic) sampling method. Another was the open trial design of the self-help programme. The third limitation was the difficulty of recruiting a large sample of healthy controls due to the lack of incentives for them to initiate the programme for health assessment. In addition, there was a high attrition rate, although the majority of participants used the programme for at least a month. High attrition rates are common in studies using self-help treatments for eating disorders (0–69.7%) [47] and natural in Internet-based trials, particularly those with self-help applications [48]. The last limitation was the demographic differences between the disordered eating participants and the healthy controls. The former group was mostly Australian, whereas the latter group was mostly Chinese. This difference may not have had much impact on the findings of this study. Recent studies identified that the eating disorder psychopathology was similar between westernised Chinese and Australians [49,50], and the clinical presentation of eating disorders in Hong Kong Chinese has increasingly been found to conform to that in Western countries [51].

Future studies using the Smart Eating programme are recommended. The effectiveness of the programme in enhancing quality of life in individuals with eating disorders could be further evaluated in a randomised controlled study. Also, the effect of the programme could be compared with specialist treatment only and the condition of combining both specialist treatment and the programme.

## Conclusions

Eating disorders have a significant impact on individuals’ quality of life. Sufferers of eating disorders report significantly poorer quality of life than healthy controls in all physical and mental health domains. This poor quality of life can be associated with eating disordered behaviours, symptom severity, depression and anxiety. The Smart Eating online self-help programme developed in the Asia-Pacific region for people with eating disorders has the potential benefit of engaging individuals in self-help behaviour and treatment, and improving their quality of life. It could be a useful adjunct to professional treatment.

## Competing interests

The authors declare that they have no competing interests.

## Authors’ contributions

SL, JM and JR contributed to the design of the study and data collection. SL analysed the findings and drafted this paper. All authors read and approved the final manuscript.
